# Weight Loss in Advanced Chronic Kidney Disease: Should We Consider Individualised, Qualitative, ad Libitum Diets? A Narrative Review and Case Study

**DOI:** 10.3390/nu9101109

**Published:** 2017-10-11

**Authors:** Irene Capizzi, Luigi Teta, Federica Neve Vigotti, Giuliana Tognarelli, Valentina Consiglio, Stefania Scognamiglio, Giorgina Barbara Piccoli

**Affiliations:** 1 Department of Clinical and Biological Sciences, University of Torino (TO), 10100 Torino, Italy; irene.capizzi@gmail.com (C.I.); g.tognarelli@samluigi.piemonte.it (T.G.); valentinaconsiglio@yahoo.it (C.V.); stescog@yahoo.it (S.S.); 2 Bioimis Accademia Alimentare, Bassano del Grappa (VI), 36061 Vicenza, Italy; tetaluigi@gmail.com; 3 Nephrology, Chivasso Hospital (Torino), 10034 Turin, Italy; fedesnow@inwind.it; 4 Néphrologie, Centre Hospitalier Le Mans, 72000 Le Mans, France

**Keywords:** chronic kidney disease, obesity, wait-listing for kidney transplantation, dialysis, weight loss, coach assisted diet, ad libitum diet

## Abstract

In advanced chronic kidney disease, obesity may bring a survival advantage, but many transplant centres demand weight loss before wait-listing for kidney graft. The case here described regards a 71-year-old man, with obesity-related glomerulopathy; referral data were: weight 110 kg, Body Mass Index (BMI) 37 kg/m^2^, serum creatinine (sCr) 5 mg/dL, estimated glomerular filtration rate (eGFR) 23 mL/min, blood urea nitrogen (BUN) 75 mg/dL, proteinuria 2.3 g/day. A moderately restricted, low-protein diet allowed reduction in BUN (45–55 mg/dL) and good metabolic and kidney function stability, with a weight increase of 6 kg. Therefore, he asked to be enrolled in a weight-loss program to be wait-listed (the two nearest transplant centres required a BMI below 30 or 35 kg/m^2^). Since previous low-calorie diets were not successful and he was against a surgical approach, we chose a qualitative, ad libitum coach-assisted diet, freely available in our unit. In the first phase, the diet is dissociated; he lost 16 kg in 2 months, without need for dialysis. In the second maintenance phase, in which foods are progressively combined, he lost 4 kg in 5 months, allowing wait-listing. Dialysis started one year later, and was followed by weight gain of about 5 kg. He resumed the maintenance diet, and his current body weight, 35 months after the start of the diet, is 94 kg, with a BMI of 31.7 kg/m^2^, without clinical or biochemical signs of malnutrition. This case suggests that our patients can benefit from the same options available to non-CKD (chronic kidney disease) individuals, provided that strict multidisciplinary surveillance is assured.

## 1. Background

The obesity pandemic is spreading worldwide [1,2,3,4,5]. Obesity is not only a major cardiovascular risk factor, but also an important element in the development of a wide range of chronic kidney diseases (CKD), including nephroangiosclerosis, and focal segmental glomerulosclerosis and diabetic nephropathy; the relationship between diabetes and obesity is so close that it has led to the coining of the word “diabesity” [6,7,8,9,10,11,12,13]. 

The pathogenesis of CKD in obese patients is complex. It can include glomerular hyperfiltration, chronic inflammation, derangements in lipid metabolism, intraglomerular hypertension and sclerosis [6,7,8,9,10,11,12,13].

While obesity represents a progression factor for all kinds of CKD, a specific picture, called obesity-related glomerulopathy (ORG), has recently been described. Its features include glomerulomegaly and the perihilar variant of focal segmental glomerulosclerosis (FSGS); from a clinical point of view, nephrotic proteinuria is common, while full-blown nephrotic syndrome is rare. Progression to kidney failure may occur, even in the absence of other lesions and diseases [14,15,16,17,18]. 

The importance of obesity in the pathogenesis of CKD is so high that the 2016 World Kidney Day was dedicated to this issue [6]. 

Obesity has a dual meaning in advanced CKD. On one hand, when dialysis is started, clinicians and patients are faced with what is called the obesity paradox—a survival advantage in obese patients on dialysis [19,20,21,22]. The overall obesity paradox is due to contrasting long-term and short-term consequences of obesity—while obesity increases long-term cardiovascular mortality, it may attenuate short-term mortality associated with malnutrition, inflammation, and protein–energy wasting [19,20,21,22].

On the other hand, this epidemiological paradox leads to a clinical paradox with respect to kidney transplantation. The transplantation issue is particularly complex; indeed, obesity significantly increases the risk of mortality and graft loss, as compared to patients with normal weights. Furthermore, the risk of surgical and post-surgical problems is increased, including retarded wound healing and infection, lymphocele, new onset diabetes after transplant, and cardiovascular disease [23,24]. The issue is important to the point that even if formal contraindications are seldom posed in the current guidelines, some transplant centres systematically choose not to wait-list obese patients [25,26,27,28,29,30,31].

However, the impact of obesity on patient survival after kidney transplantation remains somewhat controversial; while results of transplantation in obese patients may be less favorable than in patients with a normal body weight, transplanted obese patients share a similar advantage to non-obese patients with respect to dialysis. Partly as a consequence of these conflicting data, policies are not homogeneous and “medically controlled” weight loss is often required as a pre-requisite for wait-listing [24,25,26,27,28,29,30,31,32,33]. In such a setting, many experts hold that obesity should not constitute an absolute contraindication to transplantation, and that individualized risk assessment is necessary [23,24].

The controversy about weight loss is still open—the arguments in favour of having patients lose weight before kidney transplantation include the clinical plausibility of the advantage of weight loss in dialysis patients, in keeping with what is observed in the overall population, the obvious advantage of a shorter hospital stay, and lower costs of transplantation in non-obese versus obese patients. From an ethical point of view, having to cope with organ shortages suggests adopting a policy of careful allocation to those who would benefit most from transplantation [34]. However, losing weight may be difficult, if not impossible, for many patients, thus retarding or blocking their access to a treatment which is associated with an overall survival advantage [35]. 

## 2. Several Points Need to Be Clarified

The equivalent rates of survival after transplantation in obese and non-obese patients may reflect differences in selection criteria. Obese patients are more carefully selected from a cardiovascular point of view, and are only accepted in some committed centres, thus introducing a potentially relevant centre effect [34,35,36]. 

Weight loss on dialysis has been associated with lower rates of survival; however, few studies have distinguished between intentional and non-intentional weight loss and, especially in early dialysis follow-up, rapid weight loss may be a sign of poor pre-dialysis care and of cardiac disease, which are both associated with lower survival rates. A further significant factor is that surgical procedures to induce weight loss are not devoid of risk and, as a consequence, to cite the moderators’ views in the controversy over making weight loss a requirement for transplantation—recently published in Nephrology Dialysis and Transplantation—our approach should be to cautiously evaluate all the factors involved (“cum grano salis”). The problem is, in every sense, a weighty one, and each patient’s needs deserve to be taken into consideration and addressed [36].

It is in this context that we will present a short and pragmatic narrative review of the weight-loss options for CKD patients waiting for kidney transplantation. 

We also see this as an opportunity to contextualise a case treated in the pre-dialysis phase, using a relatively unexplored approach, based on having the patient follow a qualitative, ad libitum coach-assisted diet (i.e., the patient had an individual counsellor who provided encouragement and advice and ensured compliance with dietary restrictions), which had previously been tested in a small cohort of dialysis patients [37].

## 3. The Case: Presentation of the Case and Problems Related to It

A 71-year-old man, affected by stage 4 CKD, was referred to our nephrology unit in June 2013 for the prescription of a low-protein diet. He had been overweight since adolescence, and over the years his body mass index (BMI) had ranged from 35 to 40 kg/m^2^. At referral to our unit, he weighed 110 kg and had a BMI of 37 kg/m^2^.

The first detection of urinary abnormalities had occurred ten years before, when he underwent a subtotal parathyroidectomy for primary hyperparathyroidism. On the same occasion he was found to be hypertensive and anti-hypertensive treatment was started. 

The clinical picture of obesity, mild hypertension concomitant with the first signs of kidney disease, sub-nephrotic proteinuria and obesity suggested obesity-related glomerulopathy. A kidney biopsy was not proposed at referral, as it was unlikely to change clinical management of the case. At referral serum creatinine (sCr) was 5 mg/dL, estimated glomerular filtration rate (eGFR), calculated using the CKD-EPI formula, was 23 mL/min, blood urea nitrogen (BUN) was 75 mg/dL, with sub-nephrotic proteinuria (2.3 g/day). The patient was started on the moderately restricted low-protein diet we generally prescribe (a plant-based diet with protein intake of 0.6 g/kg/day, supplemented with alpha-ketoacids, and amino acids, with two unrestricted meals per week, as elsewhere described in detail [38,39]). 

For obese patients we usually calculate an average between ideal and real body weights to determine the protein intake—in fact, targeting to real weight would lead to a high protein intake, while targeting to the ideal weight is often an unreachable goal.

Compliance was good and there was an initial reduction in BUN (45–55 mg/dL), and relatively good metabolic and kidney function stabilities. One year later, sCr was 6 mg/dL, BUN was 60 mg/dL, calcium was 9.6 mg/dL, and phosphate was 5.1 mg/dL, while proteinuria ranged from 2 to 3 g per day, with normal serum albumin (4.4 g/dL) and no electrolyte imbalances. However, as the diet is based on carbohydrates, his body weight had increased by 6 kg, producing a BMI of 38.8 kg/m^2^. 

The patient had originally opted for haemodialysis, and an Arterio venous (AV) fistula was planned. Since he found dialysis severely intrusive in his everyday life and with his work as a consultant, which frequently involved travelling in Italy, he asked to be waitlisted for kidney transplantation. Since one of the two nearest transplant centres required that BMI be below 35 kg/m^2^, and the other centre only waitlisted patients whose BMI was under 30 kg/m^2^, the patient also asked to be enrolled in a weight-loss program. 

## 4. Several Options Were Explored

The patient reported that he had followed a classic low-calorie diet several times, had lost weight only for short periods, and rapidly regained it. He was against a surgical approach, in part, on account of the lack of a local referral group with specific experience with pre-dialysis and dialysis patients. Given our group’s favourable experience with a coach-assisted qualitative program for weight loss (Bioimis Accademia Alimentare), we enrolled him in the study called “RENIRE” (proteggere il RENe col dimagrIRE: protecting the kidney by losing weight; ethical committee approval: 216-2012 san Luigi Hospital). Within the setting of this study, the patient was given free consulting sessions with a dietician (Coaching Bioimis^®^).

Before starting the diet, because of the risk of rapid reduction in kidney function after discontinuation of the low-protein diet and in the hypercatabolic phase linked to weight loss, a proximal artero-venous fistula was prepared, and the patient started to be evaluated for kidney transplantation. This included a cardiology assessment, both to secure the diet phase and to control for the absence of major contraindications for transplantation. 

No sign of severe cardiac disease was found, while echocardiography demonstrated left ventricular hypertrophy, Ejection fraction (EF) 58%, slight atrial enlargement and increased myocardial mass.

## 5. The Case: The Diet 

The diet is divided into two phases: rapid weight loss and slower weight-loss and maintenance. In the first phase, the foods are selected by the nutritional coach on the basis of their glycemic index, biochemical properties and on the effect they have on increasing or decreasing the patient’s weight (data are recorded every day, to identify the most favourable “menus”). In this phase, the mean carbohydrate intake is 10–15%, protein intake is 25–30%, lipid intake is 60%, and the minimum energy intake is about 1000 kcal/day. On average, the amount of protein per kg of body weight in this phase is 1.1–1.2 g/kg/day, though the variability is high, on the account of the different meals, and the free size of the portions (*ad libitum*). In fact, in this first phase, the diet is dissociated, and only one or two foods, in unrestricted quantities are allowed at each meal. 

In the maintenance phase, foods are combined together eventually making an overall Mediterranean menu (mean intake: carbohydrates 30–50%, proteins 20% corresponding to about 0.8–1 g/kg/day, lipids 30–50%). In both phases, the diet is alcohol free and the patient is not allowed to add sugar or, in general, salt; in severe CKD; however, salt reduction is less extreme, and is tailored to each patient. Generous amounts of extra virgin olive oil can be used and there are no limits on adding spices or herbs. Examples of the different menus are presented in Table 1, Table 2 and Table 3.

At the start of the diet, blood pressure was well controlled (115/70 mmHg); body bio-impedance, performed with BCM Fresenius Kabi, showed moderate hyper-hydration of +2.8 L and a lean tissue index of 17.9 kg/m^2^ and fat tissue index of 19.4 kg/m^2^; the main anthropometric measures, the biochemical data and the changes over time for bioimpedance are reported in Table 4 and Table 5.

After discontinuation of the low-protein diet and the start of the weight-reduction program, there was a sharp decrease in the patient’s residual renal function, probably linked to the restricted sodium intake (80–100 mEq/day) and relative dehydration (as also witnessed by the presence of alkalosis). Dialysis was not needed, due to his overall well-being, good blood pressure control, preserved albumin and haemoglobin levels, and electrolyte balance, in spite of the increase in proteinuria, probably linked to the discontinuation of protein restriction (Table 5). The patient lost 16 kg in this phase (Table 4). 

In the first maintenance phase, a further 4 kg were lost in 5 months, followed by moderate weight gain, and then stabilization at 14 kg less than his pre-diet weight. This made it possible for him to be waitlisted for kidney transplantation in the first centre before starting dialysis.

A maintenance vegetable-based menu was then tailored to the patient so that dialysis could be delayed as long as possible. Dialysis was started one year later, when kidney function further deteriorated, with the development of resistant hyperphosphatemia, although in the absence of uremic symptoms and acidosis—at the first dialysis session, sCr was 11.5 mg/dL, BUN was 107 mg/dL, phosphate was 7 mg/dL, potassium was 5.5 mmol/L, serum albumin was 4.1 g/dL and haemoglobin was 10.4 g/dL.

After the first dialysis phase, in which he regained about 5 kg in 4–6 months, he started the maintenance diet again, to reach the weight required by the second transplant centre (BMI below 30 kg/m^2^). His current body weight, 35 months after the start of the diet, is 94 kg, with a BMI of 31.7 kg/m^2^; his recent bioimpedance data showed hydration of −3.1 L, lean-tissue index of 14.1 kg/m^2^ and fat-tissue index of 17.9 kg/m^2^. In the last phases of follow-up, adipose tissue mass increased slightly, while lean tissue mass decreased. This was probably due to several factors, including the age-related muscle atrophy (the patient being at that time, 74 years old), decrease in physical activity after the start of dialysis and the treatment’s catabolic effects, in spite of good dialysis efficiency (Kt/V 1.3–1.4, according to Daugirdas 2).

## 6. Discussion

### 6.1. The Paradox of Losing Weight in the Pre-Dialysis Phase

The paradox of longer survival of obese patients in dialysis—the paradigm of reverse epidemiology—underlines the importance of maintaining good nutritional status while on dialysis; hence, many authors hold that weight loss on dialysis is dangerous, failing to consider the fact that most of the large studies that have been done do not distinguish between intentional and unintentional weight loss in dialysis patients [4,5,19,20,21,22,40]. 

The unclear dangers of weight loss have to be balanced against the very real advantages of being eligible for kidney transplantation, a treatment that has a well-recognised survival advantage, also shared by overweight and obese patients [25,26,27,28,29,30,34,35,36]. While this balance would shift if policies on transplantation for obese patients were more liberal, at present losing weight is often the only way for an obese patient to get waitlisted for this treatment [34,35,36].

Conversely, losing weight in the pre-dialysis phase is sometimes reported to slow the progress of kidney disease. This is difficult to evaluate, however, as most trials and series concern relatively early CKD stages, in which the advantage of weight loss may counterbalance the negative effects dieting has on nutritional status [41,42,43]. Even less is known about the last phases of CKD. In these phases, losing weight allows patients to be waitlisted for kidney transplantation, but weight-loss induced hyper-catabolism and dialysis contribute to impairments in nutritional status [43,44]. Being hyper-catabolic can have a negative effect on a patient’s reduced kidney function, and there is a significant risk that this will lead to hyperkalaemia, hypophosphatemia and acidosis [45,46]. Furthermore, which dietary approach is most successful in allowing pre-dialysis and dialysis patients to lose weight has not yet been established. The recent consensus statement from the Asian Pacific Society of Nephrology (APSN) underlines the extent to which we lack evidence showing how the dietetic management of overweight and obese individuals with CKD should be approached. While the conclusions reached are rather disheartening, and no real consensus is reached on most of the items discussed, the fact that the APSN statement notes that medical or surgical strategies to facilitate weight loss “require a multidisciplinary approach with the involvement of a skilled renal dietician” indirectly demonstrates the importance of a tailored approach to each CKD patient [41]. 

Our patient’s story highlights the difficulties inherent in this phase, in particular, the need for careful control in the phase of rapid weight loss, so that acidosis, hyperkalemia and a rapid decrease in renal function can be avoided. 

### 6.2. Low-Protein Diets and Weight Loss

In our patient, it was almost impossible to disentangle the effects on kidney function and proteinuria caused by discontinuation of his vegetable-based, supplemented low-protein diet and those due to weight loss. Our hypothesis is that the increase in proteinuria was related to the discontinuation of his supplemented vegetable-based diet, which we believe led to a reduction in proteinuria [47,48,49]. Conversely, we thought that the rapid decrease in kidney function was mainly due to sodium restriction and relative hypotension (Table 5). In keeping with this interpretation, kidney function later stabilised and the patient was able to remain dialysis-free for over a year. 

A protein-restricted diet is usually considered to be incompatible with a calorie-restricted or weight-loss diet. Yet interestingly, when combining several Medical Subject Headings (MESH) and free terms on obesity, protein restriction, weight loss and CKD we found no relevant reports on Medline. This issue was not even commented on in APSN’s best practice statement, while the association of the two nutritional approaches was contraindicated in a recent statement by the Italian Society of Nephrology [41,50].

Our case suggests that, in selected patients, the two approaches can be combined without producing short-term detrimental effects. However, we cannot exclude that the moderate reduction in lean-body mass observed in our patient at the end of follow-up, that we considered as probably due to the hypercatabolic effect of dialysis, was at least partly due to the vegetable-based, supplemented diet he had followed in the weight-control maintenance phase, before starting dialysis (Table 4 and Table 2). 

### 6.3. Calorie-Restricted Diets in Advanced CKD

Controlling weight is difficult for all obese patients, whether or not they have CKD, and the results are often disappointing [41,50]. The classic approach of reducing calories conflicts with two of the usual indications for CKD patients: a high calorie intake to minimise the hypercatabolism linked to uraemia, acidosis, and chronic disease; and the negative effect of weight loss in patients starting renal replacement therapy [42]. 

However, the “magic numbers” of 30–35 kcal/kg day, that are usually indicated for uremic patients, are derived from a small number cases, studied over four decades ago, in a completely different population (younger, with a lower prevalence of diabetes and obesity). While the overall indications are a matter of discussion, there is widespread agreement that they do not apply to obese patients [43,49,50,51]. Furthermore, no indication of the “ideal” calorie intake in this population is available, and, consequently we lack a precise idea of what the calorie restrictions imposed should be. 

While calorie restriction of whatever entity is often beneficial for CKD patients, the fact that they are doing little physical activity and the need to conciliate a lowered calorie intake with other restrictions—which in advanced CKD and dialysis include limiting potassium and phosphate—obviously makes designing an adequate diet more complicated. Our patient had gone on traditional calorie-restricted diets several times but had never managed to maintain weight loss, a situation that is quite common in obese patients. Hence, he was reluctant to start a new diet based on the same principles. We agreed, as we felt that this approach, albeit potentially effective in patients who have never attempted to lose weight in this way, was unlikely to be of help in his case (previous failure, reduced physical activity, limited applicability of high-protein diets, due to CKD, hyperphosphatemia and acidosis, potential limitations on plant-based regimens due to the risk of hyperkalaemia).

### 6.4. Bariatric Surgery in Advanced CKD

Interest in bariatric surgery in CKD patients is also an indirect marker of the frequent failure of other means for them to attain stable weight loss [52,53,54,55,56,57]. 

The data on bariatric surgery are contrasting: weight loss is often attained, with positive results for kidney function, including reductions in hyperfiltration and proteinuria, and better blood pressure control, in virtually all CKD stages, including dialysis [56]. Studies on the effect of bariatric surgery in the strict pre-dialysis phase are lacking, but it is conceivable that the post-surgery reduction in hyperfiltration, together with the metabolic charge linked to the rapid catabolic phase, may catalyse the need for dialysis start. 

Weight loss is not without a price. While in carefully selected patients, mortality is low, and post-surgical morbidity does not appear to be higher than in the obese population without CKD, and access to kidney transplantation is becoming a specific indication, long-term effects may be important. Kidney stones, and, rarely, oxalate nephropathy, have been described in grafted kidneys [53,57].

Pancreatitis, malnutrition and malabsorption of some drugs are further concerns in selected cases. Side effects are more common in patients who have undergone an “omega” or classic bypass, and may be of lesser relevance in patients who have undergone a sleeve gastrectomy, which has been proposed by some groups, specifically to ease access to kidney transplantation [53,58,59,60,61]. 

Independently from the technique, and acknowledging the good results that can be obtained, one of the main clues to whether it will be successful is the availability of an experienced referral group with multidisciplinary experience with CKD patients. This is not yet always an option, thus limiting *de facto* access to these treatments.

### 6.5. Coach-Assisted and ad Libitum Diets

As clinicians working with obese CKD patients became aware of the shortcomings involved in classic calorie-restricted diets, different approaches were developed, including qualitative, ad libitum diets, in which unrestricted quantities of specific aliments are allowed, often in a dissociated way (i.e., one aliment at a time), and coach-assisted diets, in which compliance is reinforced by a close relationship with one individual (the coach) who acts as a counsellor and checks that the diet is being followed [62,63,64,65,66,67,68,69,70].

While these approaches have gained attention, in particular in consolidating long-term results, nephrologists’ experiences with obese CKD patients are extremely limited, possibly on account of the tendency to be more conservative when treating obese patients affected by challenging clinical problems, such as CKD. The patient discussed here was successfully treated using a coach-assisted, ad libitum, quantitative diet, following a previous positive experience with a small group of dialysis patients, in which this non-conventional approach was used mainly so that they could be waitlisted for kidney transplantation [37,71]. While these few cases do not allow us to draw conclusions about such diets’ long-term efficacies (an issue that is being addressed by a large on-going study), these “extreme” cases serve to reassure us that these approaches are without severe intrinsic side effects, and more generally suggest that no approach used with obese non-CKD patients should be banned a priori in treating obese patients with CKD. 

## 7. Conclusions

This case report reflected on the different approaches used to promote weight loss in patients with advanced CKD. While the controversy over the advantages of weight loss and making it a requirement for being waitlisted for kidney transplantation is still open, this case suggests that treatment should not be limited to traditional approaches, and that our patients can benefit from the same options available to non-CKD individuals, provided that strict multidisciplinary surveillance is available. 

## Figures and Tables

**Table 1:** Examples of menus in the rapid weight-loss phase.

Day	Breakfast	Lunch	Dinner
**Monday**	artichokes	swordfish	courgettes
**Tuesday**	artichokes	swordfish	courgettes
**Wednesday**	basmati rice	spinach	onions
**Thursday**	basmati rice	spinach	onions
**Friday**	broccoli	turkey breast	mushrooms
**Saturday**	broccoli	turkey breast	mushrooms
**Sunday**	spaghetti	pork fillet	asparagus

In this phase patients can eat a generous portion of each food on the menu, dressed with a liberal amount of extra virgin olive oil but without added salt.

**Table 2:** Examples of menus in the weight-maintenance phase.

Day	Breakfast	Lunch	Dinner
**Monday**	vegetable omelette	mixed salad of lettuce, shrimp, eggs, tuna fish or chicken and avocado	choice of fish (no shellfish)
**Tuesday**	strawberry mousse	pasta with courgettes or aubergines and cream	choice of meat, vegetables
**Wednesday**	unsalted bread with olive oil	pasta with vegetables	choice of fish (no shellfish)
**Thursday**	strawberries with cream and fructose	polenta with stewed rabbit and mushrooms	mixed beans with onions
**Friday**	spaghetti with olive oil and chilli pepper	Basmati rice with shrimp	mixed vegetables
**Saturday**	yogurt with hazelnut-cocoa cream and toasted nuts	Basmati rice with chopped meat, lentils and onions	choice of meat, vegetables
**Sunday**	yogurt with oat flakes, walnuts and almonds	rice with vegetables	choice of fish (no shellfish)

Note: In the maintenance phase, an ad libitum approach is followed. The approximate size of portions is indicated but patients do not need to weigh their food.

**Table 3:** Examples of menus in the second weight-loss, maintenance phase: a modified LPD-KA diet with 3 meals per week with unrestricted amounts of animal-derived proteins.

Day	Breakfast	Lunch	Dinner
**Monday**	unsalted bread with olive oil	mixed salad with lettuce, carrots, eggs, avocado and soybean sprouts	mixed beans with onions
**Tuesday**	strawberries with soy cream	whole wheat pasta with vegetables and soy cream	choice of fish (no shellfish),vegetables
**Wednesday**	unsalted bread with olive oil	pasta with vegetables	eggs, vegetables
**Thursday**	choice of fresh fruit with mixed nuts	whole wheat pasta with chickpeas	chicken or turkey. vegetables
**Friday**	unsweetened soy yogurt with hazelnut-cocoa cream and toasted nuts	polenta with mushrooms	mixed beans with onions
**Saturday**	unsalted bread with hazelnut-cocoa cream	Basmati rice with lentils and onions	mushrooms, aubergines and onions
**Sunday**	unsweetened soy yogurt with oat flakes, walnuts and almonds	rice with vegetables	courgettes, broccoli and artichokes

LPD-KA: Low protein diet supplemented with ketoacids and aminoacids. Note: In this phase, portion sizes are specified. Patients do not need to weigh their food but should not eat more than the amount indicated in the diet (except for the 1 to 2 protein-unrestricted meals allowed each week). Keto-analogues are taken as a dietary supplement: 1 pill per 8–10 kg of body weight per day.

**Table 4:** Anthropometric data at baseline, 2 months, 5 months, 12 months and end of follow-up.

	Baseline	2 Months “Rapid Weight Loss”	5 Months “Maintenance Phase”	12 Months “Maintenance Phase with KA”	35 Months “End of Follow Up” **
**Weight (kg)**	116	99.7 ∆−16.3	95.5 ∆−20.5	102 ∆−14	94 ∆−22
**BMI (kg/m^2^)**	38.8	33.4 ∆−5.4	31.9 ∆−6.9	34.1 ∆−4.7	31.7 ∆−7.1
**Bioimpedance OH (kg/m^2^)**	2.8	1.5	1.1	5.5	−3.1
**Bioimpedance FTI (kg/m^2^)**	19.4	12.7 ∆−6.7	11.6 ∆−7.8	14.8 ∆−4.6	17.9 ∆−1.5
**Bioimpedance LTI (kg/m^2^)**	17.9	19.8 ∆1.2	19.5 ∆1.6	16.9 ∆−1	14.1 ∆−3.8
**Handgrip (kg)**	33.8	25.8	28.0	32.6	Na
**Waist circumference (cm)**	127	111 ∆−16	116 ∆−11	115 ∆−12	112 ∆−15

Bioimpedance measurement (performed with BCM Fresenius): BMI: Body Mass Index; OH: overhydratation (litre) represents the excess fluid stored almost exclusively in the extracellular volume; LTI: Lean Tissue Index (weight/height^2^) quotient of lean tissue mass/height^2^; FTI: Fat Tissue Index (weight/height^2^) quotient of adipose tissue mass/height^2^; ** Data at end of follow up refers to pre dialysis session.

**Table 5:** Biochemical data at baseline, 2 months, 5 months, 12 months and end of follow-up.

	Baseline	2 Months “Rapid Weight Loss”	5 Months “Maintenance Phase”	12 Months “Maintenance Phase with KA”	35 Months “End of Follow Up” *
**sCr** (mg/dL)	**6.2**	**7.8**	**8.6**	**7**	**8.5**
**eGFR** CKD-EPI	**8**	**6**	**6**	**7**	**NA (dialysis)**
**BCrC** (mL/min)	**16**	**15**	**11**	**11**	**NA (dialysis)**
**BUN** (mg/dL)	**60**	**94.8**	**98.6**	**93.9**	**74.8**
**Proteinuria** (g/day)	**2.1**	**4.2**	**2.6**	**3.6**	**NA (dialysis)**
**Sodiuria** (mmol/day)	**228**	**231**	**117,5**	**170,5**	**NA (dialysis)**
**Hb** (g/dL)	**11**	**11.8**	**11.1**	**10.7**	**10.0**
**Calcium** (mg/dL)	**8.9**	**8.3**	**9.3**	**9**	**10.2**
**Phosphate** (mg/dL)	**4.4**	**5.2**	**5**	**5.9**	**4**
**iPTH** (pg/mL)	**314**	**761**	**358**	**330**	**93**
**HCO_3_** (mmol/L)	**25.6**	**32.3**	**22.3**	**24**	**21.5**
**Serum Albumin** (mg/dL)	**4.4**	**4.3**	**4.4**	**3.8**	**3.5**

sCr: serum creatinine; BCrC: creatinine clearance on 24-h urine collection; eGFR: estimated glomerular filtration rate calculated using CKD-EPI formula; BUN: blood urea nitrogen; Proteinuria: proteinuria excretion on 24-h urine collection; Hb: serum haemoglobin; iPTH: intact parathyroid hormone; HCO_3_: serum bicarbonate. * Data at end of follow-up refers to start of dialysis.
